# Stachyose Alleviates Corticosterone-Induced Long-Term Potentiation Impairment *via* the Gut–Brain Axis

**DOI:** 10.3389/fphar.2022.799244

**Published:** 2022-03-10

**Authors:** Yan Huang, Dong Li, Chen Wang, Na Sun, Wen-Xia Zhou

**Affiliations:** State Key Laboratory of Toxicology and Medical Countermeasures, Beijing Institute of Pharmacology and Toxicology, Beijing, China

**Keywords:** stachyose, corticosterone, long-term potentiation, d-serine, Bacteroidetes, Deferribacteres

## Abstract

Stress can induce learning and memory impairment; corticosterone is often used to study the effects and mechanisms of stress in animal models. Long-term potentiation (LTP) has been widely used for tackling the mechanisms of memory. Liuwei Dihuang decoction-active fraction combination (LW-AFC) can improve stress-induced LTP and cognition impairment; stachyose is an oligosaccharide in LW-AFC. The effects and mechanisms of stachyose on stress are unknown. In this study, stachyose showed protective effects against LTP impairment by corticosterone *in vivo* only *via* intragastric administration for 7 consecutive days, but there was little effect even after direct intracerebroventricular injection; the protective effect of stachyose could be canceled by non-absorbable antibiotics (ATB) which disturbed gut flora. 16S rRNA sequencing, alpha diversity, and principal coordinate analysis (PCoA) revealed that the gut flora in corticosterone-treated mice was disturbed and stachyose could improve corticosterone-induced gut flora disturbance. Bacteroidetes were decreased and Deferribacteres were increased significantly in corticosterone-treated mice, and stachyose restored Bacteroidetes and Deferribacteres to the normal level. D-serine, a coactivator of NMDA receptors, plays an important role in synaptic plasticity and cognition. Here, corticosterone had little effect on the content of D-serine and L-serine (the precursor of D-serine), but it reduced the D-serine release-related proteins, Na^+^-independent alanine–serine–cysteine transporter-1 (ASC-1), and vesicle-associated membrane protein 2 (VAMP2) significantly in hippocampus; stachyose significantly increased ASC-1 and VAMP2 in corticosterone-treated mice, and ATB blocked stachyose’s effects on ASC-1 and VAMP2. NMDA receptors co-agonists L-serine, D-serine, and glycine significantly improved LTP impairment by corticosterone. These results indicated that stachyose might indirectly increase D-serine release through the gut–brain axis to improve LTP impairment by corticosterone in the hippocampus *in vivo*.

## Introduction

Stress can activate the hypothalamic–pituitary–adrenal (HPA) axis and elevate glucocorticoids in the body (cortisol in humans and corticosterone in rodents) (de Kloet et al., 2005). Glucocorticoid receptors are abundant in the hippocampus and play an important role in stress-induced cognition alteration (De Kloet et al., 1998; Kim and Yoon, 1998; Rogalska, 2010). Long-term potentiation (LTP) is widely accepted as the cellular mechanism of learning and memory (Lüscher and Malenka, 2012). Severe stress could induce cognition impairment (Diamond and Rose, 1994; Baker and Kim, 2002; Diamond et al., 2006); corticosterone is often used to modeling cognitive impairment induced by stress (de Quervain et al., 2000; Khaksari et al., 2007). LTP deficit and cognitive impairment always coexist in stress models, and LTP impairment is often considered as one of the mechanisms for stress-induced cognitive deficits (Pavlides et al., 1993; Kim et al., 2006; Howland and Wang, 2008; Aisa et al., 2009). N-methyl-D-aspartate (NMDA) receptors play critical roles both in normal synaptic functions and excitotoxicity in the central nervous system (CNS). D-serine, a coactivator of NMDA receptors, plays an important role in the brain function (Wolosker, 2018). Our recent research found that corticosterone could decrease D-serine release-related protein and Na (+) -independent alanine–serine–cysteine transporter-1 (ASC-1), causing hypofunction of NMDA receptors, which is an important reason for LTP impairment by corticosterone (Wang et al., 2021).

Prebiotic is a group of non-digestible food ingredients that “beneficially affect the host by selectively stimulating the growth and/or activity of one or a limited number of bacterial species already resident in the colon, and thus attempt to improve host health” (Gibson and Roberfroid, 1995). Oligosaccharide is a type of prebiotics and is often severed as a gut flora modulator (Gibson and Roberfroid, 1995; Davani-Davari et al., 2019). It has reported that oligosaccharides and gut flora could modulate the D-serine pathway (Savignac et al., 2013; Kawase et al., 2017) in the brain. So, oligosaccharides may affect brain functions *via* modulating gut flora.

Liuwei Dihuang decoction (LW) is a classic formula of traditional Chinese medicine (TCM) and has been used for nearly one thousand years for various diseases with characteristic features of kidney-yin deficiency, which is related to disturbance of the neuroendocrine immunomodulation (NIM) network (Zhou et al., 2016). LW consists of six botanical drugs including Dihuang [*Rehmannia glutinosa* (Gaertn.) DC.], Shanyao (rhizome of *Dioscorea polystachya* Turcz.), Shanzhuyu (fruit of *Cornus officinalis* Siebold & Zucc.), Mudanpi (root bark of *Paeonia × suffruticosa* Andrews), Zexie (rhizome of *Alisma plantago-aquatica* L.), and Fuling [scleorotia of *Wolfiporia extensa* (Peck) Ginns] in the weight ratio 8:4:4:3:3:3. The preparation of LW has been described in our previous study in detail (Cheng et al., 2007). LW-active fraction combination (LW-AFC) is obtained from LW, which contain three kinds of main active fractions: polysaccharide fraction (LWB-B, 20.3%), glycosides fraction (LWD-b, 15.1%), and oligosaccharide fraction (CA-30, 64.6%) (Wang et al., 2016a; Wang et al., 2017a), and the preparation of LW-AFC has been described in our previous study in detail (Wang et al., 2016a). Previous researches showed that rebalancing NIM network was involved in the cognitive improving effects of LW-AFC on Alzheimer’s disease (AD) animal models, including the PrP-hA*β*PPswe/PS1^ΔE9^ (APP/PS1) mouse model and senescence-accelerated mouse prone 8 (SAMP8) strain (Wang et al., 2016a; Wang et al., 2017a). As a main form of synaptic plasticity, long-term potentiation (LTP) is believed to represent the cellular correlates of learning and memory (Lüscher and Malenka, 2012), and stress or corticosterone-induced cognitive impairment are closely related to LTP deficiency (Pavlides et al., 1993; Kim et al., 2006; Howland and Wang, 2008; Aisa et al., 2009). Modulating gut flora contributes to the neuroprotective effects of LW-AFC and CA-30 (Huang et al., 2019; Wang et al., 2019; Cheng et al., 2020a). CA-30 contains many oligosaccharides; if there is an oligosaccharide that can replace CA-30 and play its effects fully that will make the quality of LW-AFC more controllable. However, the active ingredients and mechanisms of CA-30 are not well understood yet.

Stachyose, an oligosaccharide, is an ingredient of CA-30 (Cheng et al., 2020a). It remains unknown whether stachyose has neuroprotective effects. For further understanding the active ingredients and mechanisms of CA-30, in this study, we focused on the effects of stachyose, on LTP impairment by corticosterone, gut flora, and the D-serine pathway.

## Materials and Methods

### Animals

Male BALB/c mice (18–22 g) were procured from the animal center of Academy of Military Medical Sciences (AMMS). The animals were housed in plastic cages on a 12-h light/dark cycle with controlled room temperature (24–26°C) and humidity (50–60%). Mice were acclimatized to the laboratory environment for 7 days prior to experiments with free access to a standard diet of pellets and water. Institute Animal Care and Use Committee (IACUC) of National Beijing Center for Drug Safety Evaluation and Research (NBCDSER) approved all the experimental protocols.

### Drug Administration

Corticosterone (Cort, TCI chemicals, dissolved in saline with 5% ethanol) was injected subcutaneously (50 mg/kg) 60 min before LTP was induced by high-frequency stimulation (HFS), and control animals were subcutaneously injected with a vehicle (saline with 5% ethanol). For single administration, stachyose (STA, provided by Chengdu Biopurify Phytochemicals Ltd., and the purity is 99%) was intracerebroventricularly (i.c.v., 500 μg), intraperitoneally (i.p., 20 mg/kg), and intragastrically (i.g., 450 mg/kg) administered 30 min before corticosterone. For multiple administration, stachyose was administered (i.g., i.p.) for 7 days. For disturbance of gut flora, a combination of non-absorbable antibiotics (ATB, 2.5 mg/kg pimaricin, 50 mg/kg bacitracin, and 50 mg/kg neomycin dissolved in one solution) were used in this study (Verdú et al., 2006; Huang et al., 2019), and animals received ATB (i.g.) every day 30 min before stachyose administration for 7 days. D-serine (Sigma-Aldrich), L-serine (Sigma-Aldrich), and glycine (Sigma-Aldrich) were dissolved in artificial cerebrospinal fluid (ACSF) for stocking and then diluted in ACSF before use. D-serine (300 nmol per mouse in 5 μL), L-serine (300 nmol per mouse in 5 μL), and glycine (100 nmol per mouse in 5 μL) was administered (i.c.v.) 30 min before corticosterone.

### LTP Recording

The method of LTP recording was described previously (Huang et al., 2012; Huang et al., 2013; Li et al., 2016). In brief, mice were anesthetized with urethane (1.2 g/kg, i.p.) and then fitted with ear cuffs to place in a stereotaxic frame. An incision was made along the midline of the head that made the sutures visible; a stimulating electrode was placed into the perforant path, and the recording electrode was placed into the dentate gyrus. The stimulus was delivered by a stimulator (A-M Systems Model 2100), and the responses were received by an amplifier (A-M Systems Model 1800). The WinLTP program (http://www.winltp.com) was used to record and analyze data. After a 30 min baseline recording, LTP was induced by HFS (three trains 10 s apart, 250 Hz, eight 0.1 m pulses in each train) and recorded for another 60 min. The mean population spikes (PS) amplitudes during the baseline period (0–30 min) were normalized to 100%; after HFS, the relative PS amplitudes (31–90 min) were normalized relative to the baseline period at every point.

### Gut Flora Analysis

The method has been described previously (Wang et al., 2016b). In brief, about 180–220 mg fresh stool was collected from proximal colon 1 h after corticosterone administration for each mouse. After the isolation (QIAamp DNA Stool Mini Kit, 51504, Qiagen) and validation of total genomic DNA (gDNA), two universal primers 356F (5′CCTACGGGNGGCWGCAG3′) and 803R (5′GACTACHVGGGTATCTAATCC3′) were employed to amplify V3-V4 regions of bacterial 16S rRNA gene from gDNA. The libraries were sequenced on the Illumina Miseq platform (Illumina, San Diego, CA, United States) to generate 2 × 300 bp pair end sequencing reads using the standard protocol under standard conditions. The data quality control, chimera removal, operational taxonomic unit (OTU) clustering, taxonomic assignment, and principal coordinate analyses (PCoA) were performed in QIIME.

### Determination of Neurotransmitters

D-serine and L-serine were analyzed by high-performance liquid chromatography (HPLC) in this study according to a published protocol (Wang et al., 2021). In brief, hippocampus was homogenized in 20-volume methanol, centrifuged for 15 min at 15,000 g (4°C). The supernatant was stored at −20°C until use. In total, 20°μL sample solution was added into 20 μL o-phthaldialdehyde (OPA)/N-acetyl-L-cysteine (NAC) derivatization solution (30 mg OPA and 30 mg NAC dissolved in 1°ml ethanol and then diluted in 0.2 mol/ml borate buffer to a final volume of 5°ml, pH = 9.8) for 3 min on ice before injection. An Agilent reversed-phase chromatographic column (C-18, 4.6°mm × 250 mm, 5 μm) was used. The mobile phase consisted of ammonium acetate:methanol (96:4, v/v); the flow rate was set at 1.0°ml/min; an electrochemical detector was used to detect the signals of D-serine and L-serine; and the detector potential was + 0.75 V, and the sensitivity was set at 50 nA full-scale detection. The retention times for L-serine and D-serine are 33.92 and 38.11 min, respectively. The area under curve (AUC) was used to calculate the concentration.

### Western Blotting

The method has been described previously (Huang et al., 2017). In brief, 1 h after corticosterone or vehicle injection, animals were sacrificed with CO_2_ and their hippocampal samples were obtained. The hippocampal samples were homogenized in a lysis buffer (100 mg tissue in 1°ml lysis buffer), extracted by supersonic quassation, and then centrifuged at 15,000 g for 15 min (4°C). A Bradford protein assay kit (Galen Biopharm International Co., Ltd.) determined the protein concentrations. Equal amounts of protein (30 μg) were boiled in a loading buffer for 5 min before loading on an 8% SDS-polyacrylamide gel. Electrophoresis was performed at 60 V for 30 min and then 100 V for 90°min, followed by wet transfer onto a nitrocellulose membrane at 100 V for 60 min. The membrane was blocked for 60 min in blocking solution (5% non-fat dry milk) and then incubated at 4°C overnight with anti-β-actin (1:1000, Abcam), anti-ASC-1 (1:100, Abcam), and anti-VAMP2 (1:1000, Abcam). After three washes with phosphate-buffered saline (contain 0.05% Tween 20) for 30°min, the primary antibodies were detected with the horseradish peroxidase-conjugated secondary antibodies and chemiluminescent HRP substrate (Thermo Fisher Scientific). Band density values were normalized to β-actin.

### Data Analysis

The results are presented as the mean ± SEM. GraphPad Prism 8 (GraphPad Software, Inc., United States) was used to plot and analyze the data. Student’s unpaired *t*-test was used to compare two groups. One-way analysis of variance (*ANOVA*) followed by Dunnett’s multiple comparisons test was used to compare more than two groups. *p* < 0.05 were considered statistically significant.

## Results

### The Effects of Stachyose on LTP Impairment by Corticosterone

**FIGURE 1:**
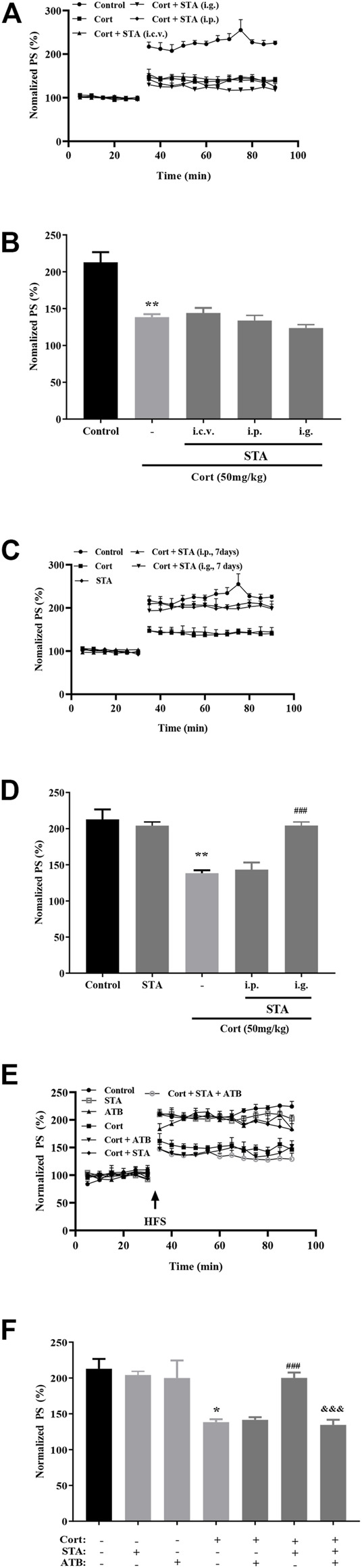
Effects of stachyose (STA) on LTP impairment by corticosterone (Cort). **(A)** Time course of average relative PS amplitudes. **(B)** Average relative PS amplitudes after HFS. Cort/vehicle was injected 60 min before HFS, and STA were administrated (i.c.v., i.p., i.g.) 30 min before Cort. **(C)** Time course of average relative PS amplitudes. **(D)** Average relative PS amplitudes after HFS. STA were administrated (i.p., i.g.) for 7 days, and the last administration was 30 min before Cort. **(E)** Time course of average relative PS amplitudes. **(F)** Average relative PS amplitudes after HFS. ATB was administrated 30 min before STA (i.g.). Data are shown as the mean ± SEM, *n* = 4−6. *p < 0.05, **p < 0.01 compared to the Control group; ^###^ *p* < 0.001 compared to the Cort group; ^&&&^ *p* < 0.001 compared to the STA + Cort group.

Results of electrophysiological experiments showed that the average values of the relative PS amplitudes after HFS in the 50 mg/kg corticosterone group were significantly lower than that in the Control group, indicating that corticosterone significantly impaired hippocampal LTP. After single administration (i.c.v., i.p., and i.g.), stachyose had no protective effect on LTP impairment by corticosterone (Figures 1A,B; Cort *vs.* Control: *p* = 0.0011; STA (i.c.v.) *vs.* Cort: *p* = 0.8226, STA (i.p.) *vs.* Cort: *p* = 0.8997, STA (i.g.) *vs.* Cort: *p* = 0.2286). After intraperitoneal administration for 7 days, stachyose had no protective effect, while stachyose showed protective effect after intragastric administration for 7 days (Figures 1C,D, Cort *vs.* Control: *p* = 0.0011; STA *vs.* Control: *p* = 0.8698; STA (i.p.) *vs.* Cort: *p* = 0.8259; STA (i.g.) *vs.* Cort: *p* < 0.001).

ATB was used to disturb gut flora, results showed that administration of ATB had no effect on hippocampal LTP in control and corticosterone-treated mice. Administration (i.g.) of stachyose for 7 days significantly improved LTP impairment by corticosterone, while ATB canceled the beneficial effect of stachyose on LTP impairment by corticosterone (Figures 1E,F, Cort *vs.* Control: *p* = 0.0191, ATB *vs*. Control: *p* = 0.8174, Cort + ATB *vs*. Cort: *p* = 0.8816, Cort + STA *vs.* Cort: *p* < 0.001, Cort + STA + ATB *vs.* Cort + STA: *p* = 0.0004), suggesting that stachyose may display its protective effects *via* gut flora.

### The Effects of Stachyose on Gut Flora

**FIGURE 2:**
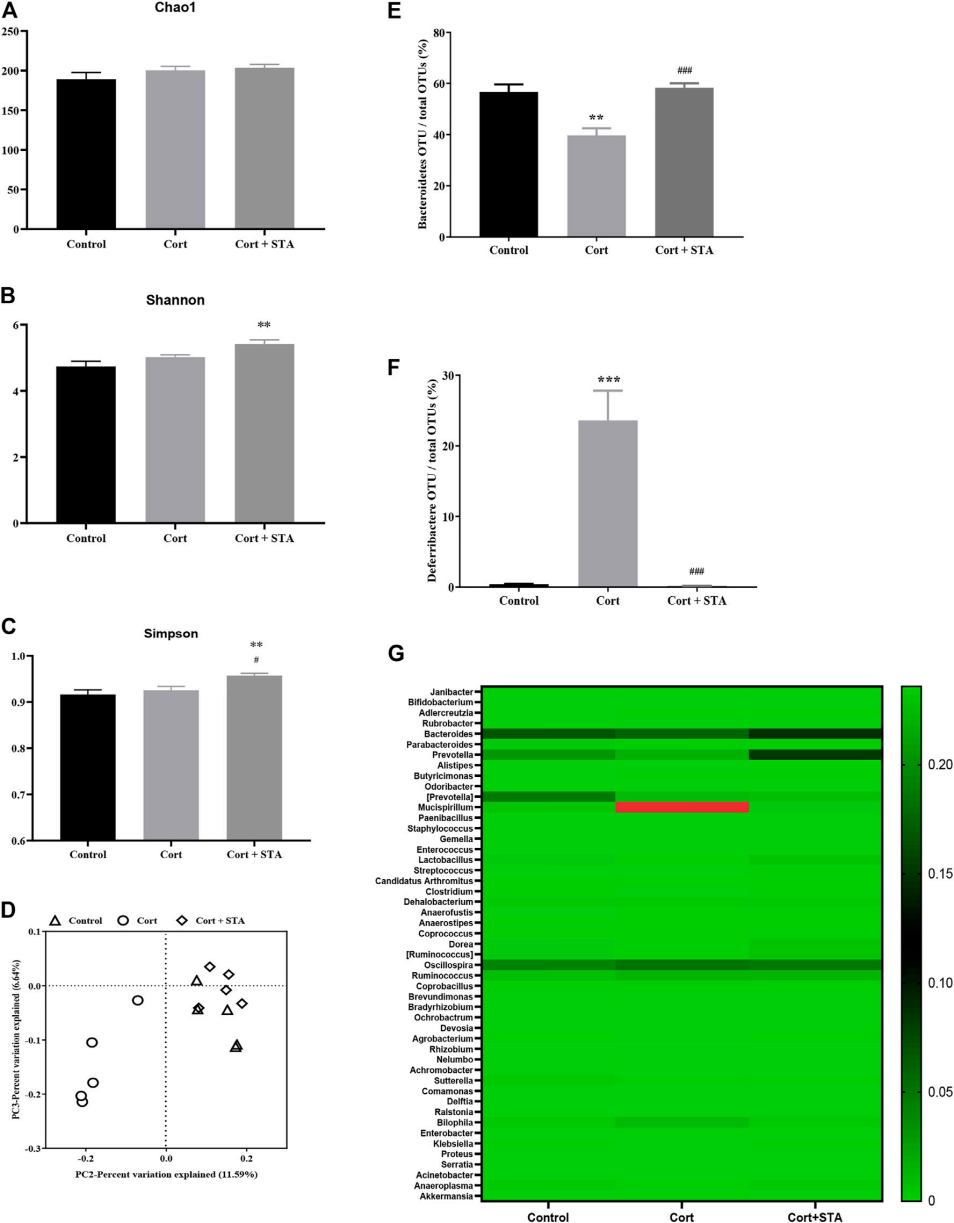
Effects of stachyose (STA) on gut flora. **(A–C)** Evaluation of the alpha diversity of gut flora. **(D)** PCA plot based on subsampled unweighted Unifrac distances. **(E)** Relative abundance of Bacteroidetes operational taxonomic unit (OTU) at the phylum level. **(F)** Relative abundance of Deferribacteres OTU at the phylum level. **(G)** Heatmap of the 49 most abundant OTUs at the genus level. Data are shown as the mean ± SEM, *n* = 5. *p < 0.05, **p < 0.01, ***p < 0.001 compared to the Control group; ^#^ *p* < 0.05, ^###^ *p* < 0.001 compared to the Cort group.

The Chao1, Shannon, and Simpson indices were evaluated to estimate the alpha diversity of gut flora. Corticosterone had little effects on these indices, and stachyose increased the Shannon and Simpson indices significantly (Figures 2A–C, Shannon, Cort *vs.* Control: *p* = 0.2679, Cort + STA *vs.* Control: *p* = 0.0049, Cort + STA *vs.* Cort: *p* = 0.0894; Simpson, Cort *vs.* Control: *p* = 0.6952, Cort + STA *vs.* Control: *p* = 0.0078, Cort + STA *vs.* Cort: *p* = 0.0341), indicating that stachyose increased the diversity of gut flora. The principal coordinate analysis (PCoA) showed that the gut flora in corticosterone-treated mice was distinct from that in the control group, and the gut flora in stachyose-treated mice clustered together with that in the control group (Figure 2D).

At the phylum level, compared with the control group, relative abundance of Bacteroidetes decreased and relative abundance of Deferribacteres increased significantly in corticosterone-treated mice, and stachyose restored Bacteroidetes and Deferribacteres to the normal level (Figure 2E, Bacteroidetes, Cort *vs.* Control: *p* = 0.0030, Cort + STA *vs.* Cort: *p* = 0.0005. Figure 2F, Deferribacteres, Cort *vs.* Control: *p* = 0.0006, Cort + STA *vs.* Cort: *p* = 0.0005). The heatmap of the 49 most abundant OTUs at the genus level (Figure 2G) showed that Bacteroides and Prevotella (belonging to Bacteroidetes at phylum level) deceased in corticosterone-treated mice, and stachyose restored them; Mucispirillum (belonging to Deferribacteres at phylum level) increased in corticosterone-treated mice, and stachyose restored them, further demonstrating that stachyose may exert moderating effect on gut flora.

### The Effects of Stachyose on D-serine Metabolic Pathway

**FIGURE 3:**
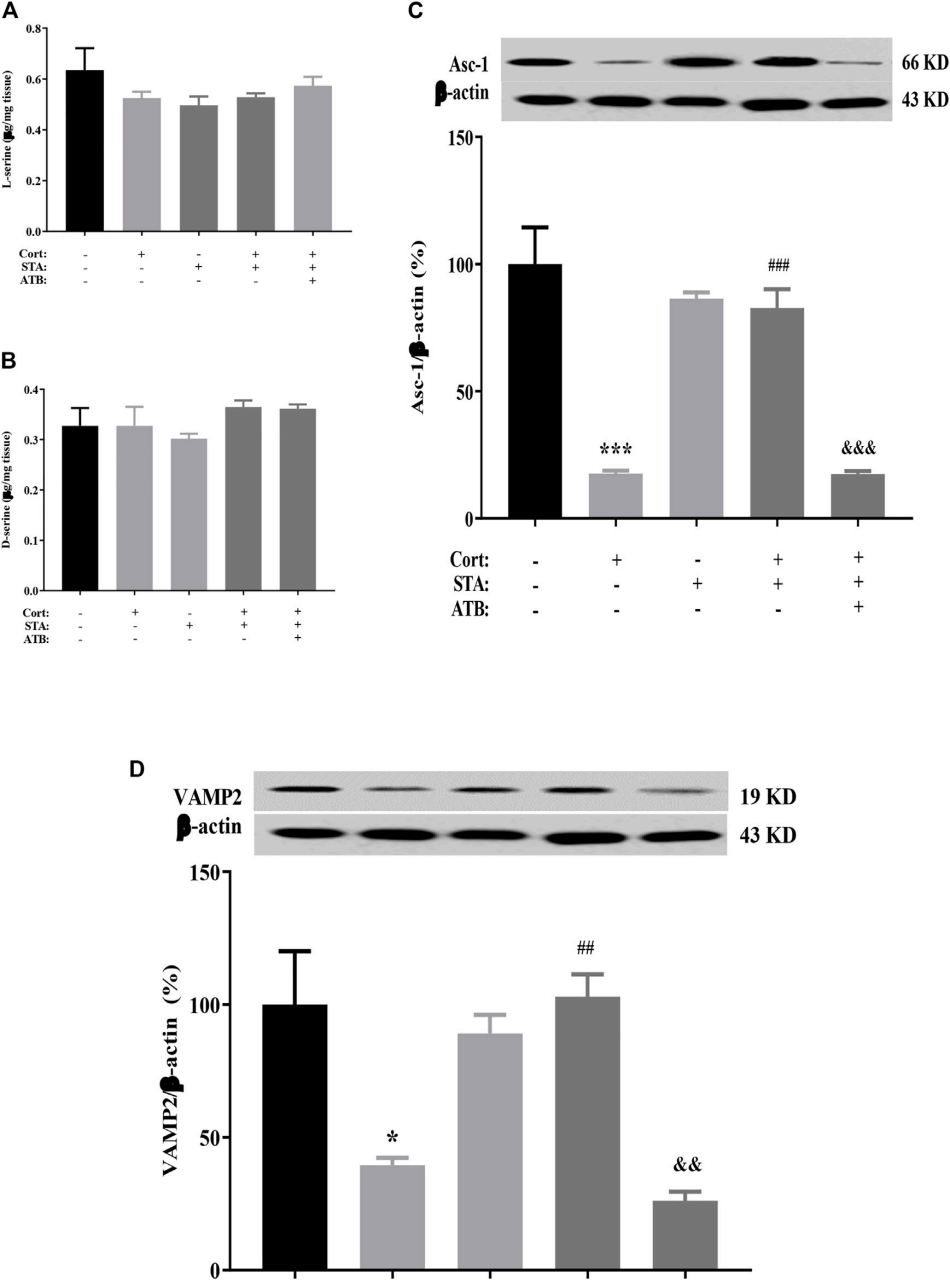
Effects of stachyose (STA) on the D-serine metabolic pathway. **(A)** Effects of STA on the level of l-serine in the hippocampus. **(B)** Effects of STA on the level of D-serine in the hippocampus. **(C)** Effects of STA on the protein expression level of ASC-1 in the hippocampus. **(D)** Effects of STA on the protein expression level of VAMP2 in the hippocampus. Data are shown as the mean ± SEM, n = 4. *p < 0.05, ***p < 0.001 compared to the Control group; ^##^ *p* < 0.01, ^###^ *p* < 0.001 compared to the Cort group; ^&&^ *p* < 0.01, ^&&&^ *p* < 0.001 compared to the STA + Cort group. ASC-1, Na + -independent alanine–serine–cysteine transporter-1; VAMP2, vesicle-associated membrane protein 2.

HPLC results showed that the content of D-serine and its precursor L-serine in the hippocampus did not change in corticosterone-treated mice. Stachyose had no effect on the level of hippocampal D-serine and L-serine in control and corticosterone-treated mice. ATB had no effect on the level of hippocampal D-serine and L-serine in stachyose and corticosterone-treated mice (Figure 3A, L-serine, Cort *vs.* Control: *p* = 0.4774, STA *vs.* Control: *p* = 0.2651, Cort + STA *vs.* Cort: *p* = 0.9949, Cort + STA + ATB *vs.* Cort + STA: *p* = 0.9454; Figure 3B, D-serine, Cort *vs.* Control: *p* = 0.9998, STA *vs.* Control: *p* = 0.9450, Cort + STA *vs.* Cort: *p* = 0.8185, Cort + STA + ATB *vs.* Cort + STA: *p* = 0.8652). However, the protein expression level of hippocampal ASC-1 and VAMP2 were significantly decreased in corticosterone-treated mice. Stachyose significantly increased the expression level of hippocampal ASC-1 and VAMP2 in corticosterone-treated mice and had no effect on the level of hippocampal ASC-1 in control mice. ATB significantly canceled the increased effect of stachyose on the protein expression level of hippocampal ASC-1 and VAMP2 in corticosterone-treated mice (Figure 3C, ASC-1, Cort *vs.* Control: *p* = 0.0009, STA *vs.* Control: *p* = 0.4631, Cort + STA *vs.* Cort: *p* = 0.0010, Cort + STA + ATB *vs.* Cort + STA: *p* = 0.0009. Figure 3D, VAMP2, Cort *vs.* Control: *p* = 0.0237, STA *vs.* Control: *p* = 0.7700, Cort + STA *vs.* Cort: *p* = 0.0020, Cort + STA + ATB *vs.* Cort + STA: *p* = 0.0011), suggesting that stachyose may restore D-serine release to improve LTP impairment by corticosterone.

### The Effects of D-Serine, L-Serine, and Glycine on LTP Impairment by Corticosterone

**FIGURE 4:**
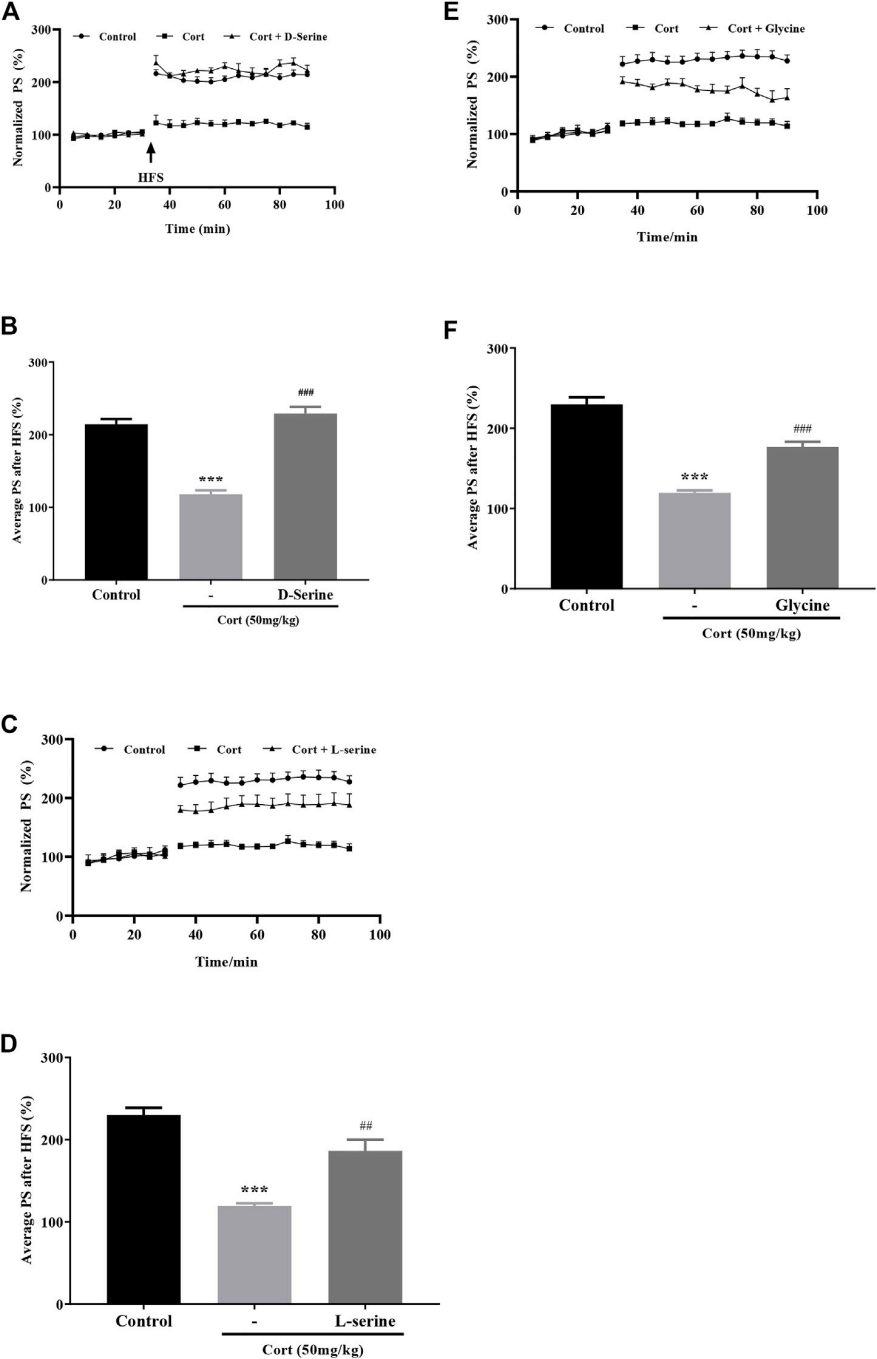
Effects of D-serine, l-serine, and glycine on LTP impairment by corticosterone (Cort). **(A)** Time course of average relative PS amplitudes. **(B)** Average relative PS amplitudes after HFS; Cort/vehicle was injected 60 min before HFS, and d-serine was administrated 30 min before Cort. **(C)** Time course of average relative PS amplitudes. **(D)** Average relative PS amplitudes after HFS; Cort/vehicle was injected 60 min before HFS, and l-serine was administrated 30 min before Cort. **(E)** Time course of average relative PS amplitudes. **(F)** Average relative PS amplitudes after HFS; Cort/vehicle was injected 60 min before HFS, and glycine was administrated 30 min before Cort. Data are shown as the mean ± SEM, *n* = 4–7. ***p < 0.001 compared to the Control group; ^##^ *p* < 0.01, ^###^ *p* < 0.001 compared to the Cort group.

Results showed that administration (i.c.v.) of endogenous NMDA receptor glycine site full agonist D-serine (300 nmol) significantly improved corticosterone-induced hippocampal LTP impairment (Figures 4A,B, Cort *vs.* Control: *p* < 0.001, Cort + D-serine *vs.* Cort: *p* < 0.001). Administration (i.c.v.) of L-serine (300 nmol), the precursor of D-serine, also significantly improved corticosterone-induced hippocampal LTP impairment (Figures 4C,D, Cort *vs.* Control: *p* < 0.001, Cort + L-serine *vs.* Cort: *p* = 0.0037). Results also showed that administration (i.c.v.) of endogenous NMDA receptor glycine site partial agonist glycine (100 nmol) significantly improved corticosterone-induced hippocampal LTP impairment (Figures 4E,F, Cort *vs.* Control: *p* < 0.001, Cort + glycine *vs.* Cort: *p* = 0.0002), demonstrating that stachyose may enhance the function NMDA receptors by restoring D-serine release to improve LTP impairment by corticosterone.

## Discussion

The dosage of stachyose is based on the dosage of LW-AFC. According to our previous study, 0.8 g/kg to 3.2 g/kg of LW-AFC (intragastric administration) could protect LTP impairment against corticosterone (Huang et al., 2019); LW-AFC contains 64.6% oligosaccharide fraction, that is, the theoretical effective dosage of oligosaccharide fraction is ranged from 520 mg/kg to 2080 mg/kg. As one of the main ingredients in oligosaccharide fraction, the effective dosage of stachyose might equal or less than that of oligosaccharide fraction. So, 450 mg/kg of stachyose (intragastric administration) will not exceed a pharmacologically meaningful level. Furthermore, the toxicity of stachyose was very low, whereas intracerebroventricularly (500 μg) and intraperitoneally (20 mg/kg) administered relative high dosage showed little effect on normal animals. Because the effects of LW-AFC and oligosaccharide fraction has been fully demonstrated (Wang et al., 2016a; Wang et al., 2016b; Wang et al., 2016c; Wang et al., 2017a; Wang et al., 2017b; Wang et al., 2017c; Huang et al., 2019; Zeng et al., 2019; Cheng et al., 2020b; Wei et al., 2021) and stachyose is one of the main ingredients in oligosaccharide fraction, we used a single effective dosage of stachyose to study the mechanisms.

In this study, single administration of stachyose *via* i.p and i.g., even direct in the brain *via* i.c.v., has little effect on LTP impairment by corticosterone. Many studies have confirmed that stachyose can regulate the gut flora balance and act as prebiotic (Li et al., 2013; Li et al., 2017; Liu et al., 2018), so stachyose would have protective effects against LTP impairment by corticosterone *via* intragastric administration for a few days. The data in this study showed that 7-consecutive-day administration of stachyose *via* i.g. had protective effect, and there was little effect *via* i.p. To disturb gut flora, a combination of non-absorbable antibiotics (ATB) (Huang et al., 2019) were applied, the results showed that ATB canceled the protective effect of stachyose without affecting LTP in control and corticosterone-treated mice, suggesting that stachyose may display its protective effects against LTP impairment by corticosterone *via* gut flora.

The alpha diversity showed that corticosterone did not change the diversity and richness of gut flora, and stachyose could increase the diversity, indicating that higher gut flora diversity and richness might contribute to the protective effect of stachyose against corticosterone. The PCoA analysis of gut flora showed that corticosterone-treated mice were distinct from mice in the control group, and the gut flora in stachyose-treated mice clustered together with that in the control group. Also, Stachyose could also restore Bacteroidetes and Deferribacteres to normal level. It has been reported that Bacteroidetes is related to cognitive development in human infants and dementia (Carlson et al., 2018; Saji et al., 2019). We found that Bacteroidetes decreased in corticosterone treated mice, which is similar to previous report (Qiu et al., 2019). Shi’s study (Shi et al., 2020) showed that restored Bacteroidetes level was related to cognitive improving effect of β-glucan. Results in this study showed that stachyose could increase the relative abundance of Bacteroidetes and restore it to normal level. Increased Deferribacteres was associated to inflammation (Berry et al., 2015), and decreased Deferribacteres level was reported to relate to cognitive improving effect of methylene blue (Gureev et al., 2020). Here we found that Deferribacteres increased significantly in corticosterone-treated mice, and stachyose could restore it to normal level. These results indicated that modulating gut flora such as Bacteroidetes and Deferribacteres might be an important factor for stachyose’s neuroprotective effect.

D-serine is synthesized by the enzyme serine racemase (SR) which is mainly localized in neurons (Benneyworth et al., 2012; Balu et al., 2014), playing an important role in brain function. Na^+^-independent alanine–serine–cysteine transporter-1 (ASC-1) and vesicle-associated membrane protein 2 (VAMP2) are responsible for the transport and release of D-serine (Mothet et al., 2005; Rosenberg et al., 2013; Neame et al., 2019). Previous studies indicated that D-serine could improve memory deficit by stress through activation of NMDA receptors (Choi et al., 1987; Guercio et al., 2014; Wang et al., 2017d; Gonçalves-Ribeiro et al., 2019), suggesting that hypofunction of NMDA receptors might an important factor for stress-induced cognitive impairment. And we recently found that corticosterone decreased ASC-1 and VAMP2, which are important for D-serine release, leading to hypofunction of the NMDA receptors and LTP impairment (Wang et al., 2021). In this study, the results showed that stachyose had no effect on the content of L-serine or D-serine in hippocampal tissue, but increased D-serine release-related proteins ASC-1 and VAMP2. Along with a combination of non-absorbable antibiotics, the effect of stachyose on ASC-1 and VAMP2 was blocked. Modulating gut flora might contribute to the effect of stachyose on the D-serine pathway. It has reported that D-serine and L-serine were lower in the brain of specific pathogen-free (SPF) mice than in those of germ-free (GF) mice, indicating that gut flora might be involved in the D-serine metabolic pathway in the brain (Kawase et al., 2017). Oligosaccharides, such as galacto-oligosaccharides (GOS), administrated *via* i.g. could increase hippocampal D-serine level (Savignac et al., 2013). D-serine is a coactivator of NMDA receptor, binding to the glycine site of NMDA receptor (Wolosker, 2018). These results indicated that increase D-serine release might contribute to neuroprotective effect of stachyose against corticosterone *via* modulating gut flora. It is a limitation that how the gut flora modulates the D-serine pathway in the brain also remains elusive.

**FIGURE 5:**
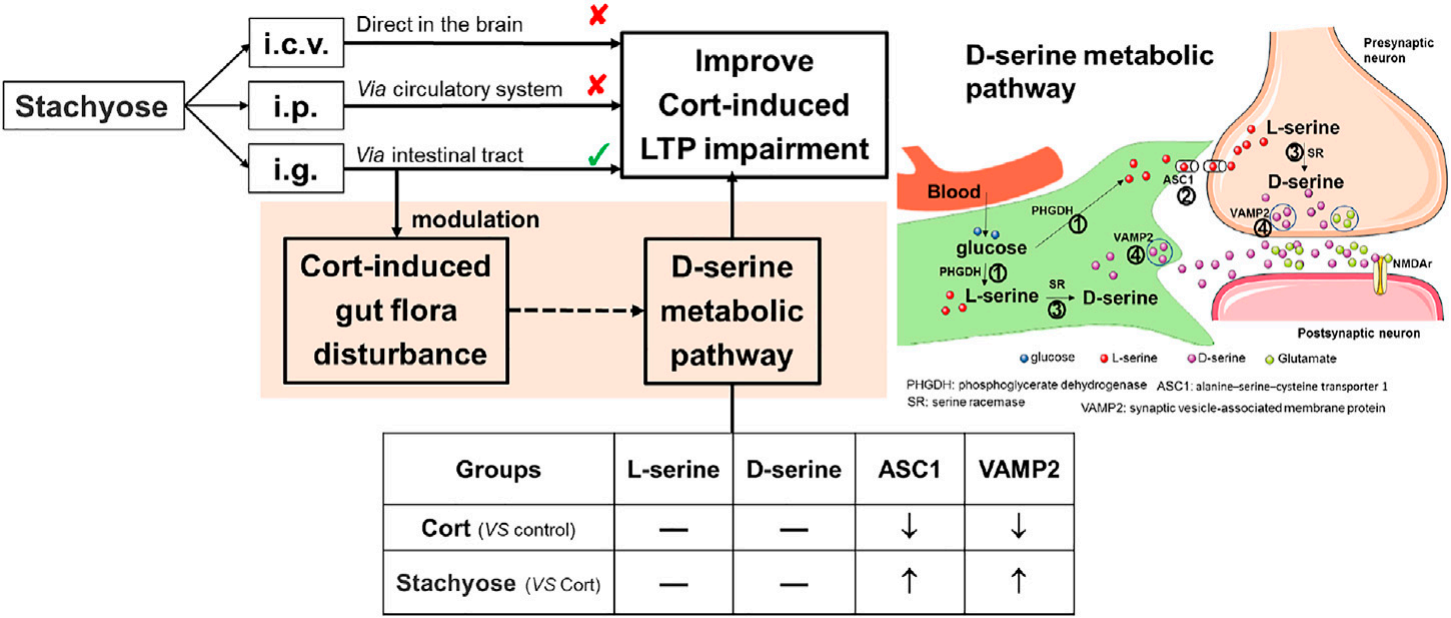
Summary of effects and mechanisms of stachyose. Stachyose can protect LTP impairment against corticosterone *via* intragastric administration, but there is little effect when administration *via* intraperitoneal injection or intracerebroventricular injection. Modulating the gut flora is involved in the beneficial effects of stachyose. Stachyose can restore the D-serine metabolic pathway disturbed by corticosterone.

Altogether, stachyose have a very similar effect on LTP impairment by corticosterone, which makes it a candidate for replacing CA-30 in LW-AFC. Stachyose could increase the D-serine release-related proteins ASC-1 and VAMP2 *via* gut–brain axis, protecting LTP from deteriorative effect of corticosterone (Figure 5). This study contributes to the understanding of active ingredients and mechanisms of LW-AFC. Further study is needed to uncover the relation between gut flora and the D-serine metabolic pathway.

## Data Availability

The sequence data have been deposited in the National Center for Biotechnology Information (NCBI) BioProject database with project number PRJNA773852. All other data are available upon request from the authors.

## Abbreviations

AD, Alzheimer’s disease; APP/PS1, PrP-hAβPPswe/PS1ΔE9; Aβ, amyloid *β*-protein; CA-30, oligosaccharide fraction of LW-AFC; Cort, Corticosterone; HFS, high-frequency stimulation; HPA, hypothalamic–pituitary–adrenal; LTP, long-term potentiation; LW-AFC, LW-active fraction combination; LWB-B, polysaccharide fraction of LW-AFC; LWD-b, glycoside fraction of LW-AFC; NIM, neuroendocrine immunomodulation; SAMP8, senescence-accelerated mouse prone 8; TCM, traditional Chinese medicine.

## References

[B1] Aisa B. Elizalde N. Tordera R. Lasheras B. Del Río J. Ramírez M. J. (2009). Effects of Neonatal Stress on Markers of Synaptic Plasticity in the hippocampus: Implications for Spatial Memory. Hippocampus 19, 1222–1231. 10.1002/hipo.20586 19309038

[B2] Baker K. B. Kim J. J. (2002). Effects of Stress and Hippocampal NMDA Receptor Antagonism on Recognition Memory in Rats. Learn. Mem. 9, 58–65. 10.1101/lm.46102 11992016 PMC155932

[B3] Balu D. T. Takagi S. Puhl M. D. Benneyworth M. A. Coyle J. T. (2014). D-serine and Serine Racemase Are Localized to Neurons in the Adult Mouse and Human Forebrain. Cell Mol Neurobiol 34, 419–435. 10.1007/s10571-014-0027-z 24436034 PMC4505359

[B4] Benneyworth M. A. Li Y. Basu A. C. Bolshakov V. Y. Coyle J. T. (2012). Cell Selective Conditional Null Mutations of Serine Racemase Demonstrate a Predominate Localization in Cortical Glutamatergic Neurons. Cel Mol Neurobiol 32, 613–624. 10.1007/s10571-012-9808-4 PMC4817353 22362148

[B5] Berry D. Kuzyk O. Rauch I. Heider S. Schwab C. Hainzl E. (2015). Intestinal Microbiota Signatures Associated with Inflammation History in Mice Experiencing Recurring Colitis. Front. Microbiol. 6, 1408. 10.3389/fmicb.2015.01408 26697002 PMC4678223

[B6] Carlson A. L. Xia K. Azcarate-Peril M. A. Goldman B. D. Ahn M. Styner M. A. (2018). Infant Gut Microbiome Associated with Cognitive Development. Biol. Psychiatry 83, 148–159. 10.1016/j.biopsych.2017.06.021 28793975 PMC5724966

[B7] Cheng X. Huang Y. Zhang Y. Zhou W. (2020). LW-AFC, a New Formula from the Traditional Chinese Medicine Liuwei Dihuang Decoction, as a Promising Therapy for Alzheimer's Disease: Pharmacological Effects and Mechanisms. Adv. Pharmacol. 87, 159–177. 10.1016/bs.apha.2019.10.005 32089232

[B8] Cheng X. Huang Y. Zhang Y. Zhou W. (2020). “LW-AFC, a New Formula from the Traditional Chinese Medicine Liuwei Dihuang Decoction, as a Promising Therapy for Alzheimer's Disease: Pharmacological Effects and Mechanisms,” in Advances in Pharmacology (Cambridge, Massachusetts, United States: Academic Press). 10.1016/bs.apha.2019.10.005 32089232

[B9] Cheng X. R. Zhou W. X. Zhang Y. X. (2007). The Effects of Liuwei Dihuang Decoction on the Gene Expression in the hippocampus of Senescence-Accelerated Mouse. Fitoterapia 78, 175–181. 10.1016/j.fitote.2006.11.006 17337329

[B10] Choi D. W. Maulucci-Gedde M. Kriegstein A. R. (1987). Glutamate Neurotoxicity in Cortical Cell Culture. J. Neurosci. 7, 357–368. 10.1523/jneurosci.07-02-00357.1987 2880937 PMC6568898

[B11] Davani-Davari D. Negahdaripour M. Karimzadeh I. Seifan M. Mohkam M. Masoumi S. J. (2019). Prebiotics: Definition, Types, Sources, Mechanisms, and Clinical Applications. Foods 8, 92. 10.3390/foods8030092 PMC6463098 30857316

[B12] de Kloet E. R. Joëls M. Holsboer F. (2005). Stress and the Brain: from Adaptation to Disease. Nat. Rev. Neurosci. 6, 463–475. 10.1038/nrn1683 15891777

[B13] De Kloet E. R. Vreugdenhil E. Oitzl M. S. Joëls M. (1998). Brain Corticosteroid Receptor Balance in Health and Disease. Endocr. Rev. 19, 269–301. 10.1210/edrv.19.3.0331 9626555

[B14] de Quervain D. J. Roozendaal B. Nitsch R. M. McGaugh J. L. Hock C. (2000). Acute Cortisone Administration Impairs Retrieval of Long-Term Declarative Memory in Humans. Nat. Neurosci. 3, 313–314. 10.1038/73873 10725918

[B15] Diamond D. M. Campbell A. M. Park C. R. Woodson J. C. Conrad C. D. Bachstetter A. D. (2006). Influence of Predator Stress on the Consolidation versus Retrieval of Long-Term Spatial Memory and Hippocampal Spinogenesis. Hippocampus 16, 571–576. 10.1002/hipo.20188 16741974

[B16] Diamond D. M. Rose G. M. (1994). Stress Impairs LTP and Hippocampal-dependent Memory. Ann. N. Y Acad. Sci. 746, 411–414. 10.1111/j.1749-6632.1994.tb39271.x 7825902

[B17] Gibson G. R. Roberfroid M. B. (1995). Dietary Modulation of the Human Colonic Microbiota: Introducing the Concept of Prebiotics. J. Nutr. 125, 1401–1412. 10.1093/jn/125.6.1401 7782892

[B18] Gonçalves-Ribeiro J. Pina C. C. Sebastião A. M. Vaz S. H. (2019). Glutamate Transporters in Hippocampal LTD/LTP: Not Just Prevention of Excitotoxicity. Front Cel Neurosci 13, 357. 10.3389/fncel.2019.00357 PMC6691053 31447647

[B19] Guercio G. D. Bevictori L. Vargas-Lopes C. Madeira C. Oliveira A. Carvalho V. F. (2014). D-serine Prevents Cognitive Deficits Induced by Acute Stress. Neuropharmacology 86, 1–8. 10.1016/j.neuropharm.2014.06.021 24978104

[B20] Gureev A. P. Syromyatnikov M. Y. Ignatyeva D. A. Valuyskikh V. V. Solodskikh S. A. Panevina A. V. (2020). Effect of Long-Term Methylene Blue Treatment on the Composition of Mouse Gut Microbiome and its Relationship with the Cognitive Abilities of Mice. PLoS One 15, e0241784. 10.1371/journal.pone.0241784 33206681 PMC7673545

[B21] Howland J. G. Wang Y. T. (2008). Synaptic Plasticity in Learning and Memory: Stress Effects in the hippocampus. Prog. Brain Res. 169, 145–158. 10.1016/S0079-6123(07)00008-8 18394472

[B22] Huang Y. Hu Z. Liu G. Zhou W. Zhang Y. (2013). Cytokines Induced by Long-Term Potentiation (LTP) Recording: a Potential Explanation for the Lack of Correspondence between Learning/memory Performance and LTP. Neuroscience 231, 432–443. 10.1016/j.neuroscience.2012.11.010 23201254

[B23] Huang Y. Li D. Cheng B. Liu G. Zhang Y. X. Zhou W. X. (2019). Active Fraction Combination from Liuwei Dihuang Decoction (LW-AFC) Ameliorates Corticosterone-Induced Long-Term Potentiation (LTP) Impairment in Mice *In Vivo* . J. Ethnopharmacol 236, 147–154. 10.1016/j.jep.2019.03.002 30851370

[B24] Huang Y. Shen W. Su J. Cheng B. Li D. Liu G. (2017). Modulating the Balance of Synaptic and Extrasynaptic NMDA Receptors Shows Positive Effects against Amyloid-β-Induced Neurotoxicity. J. Alzheimers Dis. 57, 885–897. 10.3233/JAD-161186 28269783

[B25] Huang Y. Yang S. Hu Z. Y. Liu G. Zhou W. X. Zhang Y. X. (2012). A New Approach to Location of the Dentate Gyrus and Perforant Path in Rats/mice by Landmarks on the Skull. Acta Neurobiol. Exp. (Wars) 72, 468–472. 23377276 10.55782/ane-2012-1917

[B26] Kawase T. Nagasawa M. Ikeda H. Yasuo S. Koga Y. Furuse M. (2017). Gut Microbiota of Mice Putatively Modifies Amino Acid Metabolism in the Host Brain. Br. J. Nutr. 117, 775–783. 10.1017/S0007114517000678 28393748

[B27] Khaksari M. Rashidy-Pour A. Vafaei A. A. (2007). Central Mineralocorticoid Receptors Are Indispensable for Corticosterone-Induced Impairment of Memory Retrieval in Rats. Neuroscience 149, 729–738. 10.1016/j.neuroscience.2007.08.016 17945427

[B28] Kim J. J. Song E. Y. Kosten T. A. (2006). Stress Effects in the hippocampus: Synaptic Plasticity and Memory. Stress 9, 1–11. 10.1080/10253890600678004 16753928

[B29] Kim J. J. Yoon K. S. (1998). Stress: Metaplastic Effects in the hippocampus. Trends Neurosci. 21, 505–509. 10.1016/s0166-2236(98)01322-8 9881846

[B30] Li D. Huang Y. Cheng B. Su J. Zhou W. X. Zhang Y. X. (2016). Streptozotocin Induces Mild Cognitive Impairment at Appropriate Doses in Mice as Determined by Long-Term Potentiation and the Morris Water Maze. J. Alzheimers Dis. 54, 89–98. 10.3233/JAD-150979 27472873

[B31] Li T. Lu X. Yang X. (2017). Evaluation of Clinical Safety and Beneficial Effects of Stachyose-Enriched α-galacto-oligosaccharides on Gut Microbiota and Bowel Function in Humans. Food Funct. 8, 262–269. 10.1039/c6fo01290f 28001151

[B32] Li T. Lu X. Yang X. (2013). Stachyose-enriched α-galacto-oligosaccharides Regulate Gut Microbiota and Relieve Constipation in Mice. J. Agric. Food Chem. 61, 11825–11831. 10.1021/jf404160e 24245736

[B33] Liu G. Bei J. Liang L. Yu G. Li L. Li Q. (2018). Stachyose Improves Inflammation through Modulating Gut Microbiota of High-Fat Diet/Streptozotocin-Induced Type 2 Diabetes in Rats. Mol. Nutr. Food Res. 62, e1700954. 10.1002/mnfr.201700954 29341443

[B34] Lüscher C. Malenka R. C. (2012). NMDA Receptor-dependent Long-Term Potentiation and Long-Term Depression (LTP/LTD). Cold Spring Harb Perspect. Biol. 4, a005710. 10.1101/cshperspect.a005710 22510460 PMC3367554

[B35] Mothet J. P. Pollegioni L. Ouanounou G. Martineau M. Fossier P. Baux G. (2005). Glutamate Receptor Activation Triggers a Calcium-dependent and SNARE Protein-dependent Release of the Gliotransmitter D-Serine. Proc. Natl. Acad. Sci. U S A. 102, 5606–5611. 10.1073/pnas.0408483102 15800046 PMC556243

[B36] Neame S. Safory H. Radzishevsky I. Touitou A. Marchesani F. Marchetti M. (2019). The NMDA Receptor Activation by D-Serine and glycine Is Controlled by an Astrocytic Phgdh-dependent Serine Shuttle. Proc. Natl. Acad. Sci. U S A. 116, 20736–20742. 10.1073/pnas.1909458116 31548413 PMC6789919

[B37] Pavlides C. Watanabe Y. McEwen B. S. (1993). Effects of Glucocorticoids on Hippocampal Long-Term Potentiation. Hippocampus 3, 183–192. 10.1002/hipo.450030210 8353605

[B38] Qiu D. Xia Z. Deng J. Jiao X. Liu L. Li J. (2019). Glucorticoid-induced Obesity Individuals Have Distinct Signatures of the Gut Microbiome. Biofactors 45, 892–901. 10.1002/biof.1565 31588658

[B39] Rogalska J. (2010). Mineralocorticoid and Glucocorticoid Receptors in hippocampus: Their Impact on Neurons Survival and Behavioral Impairment after Neonatal Brain Injury. Vitam Horm. 82, 391–419. 10.1016/S0083-6729(10)82020-5 20472149

[B40] Rosenberg D. Artoul S. Segal A. C. Kolodney G. Radzishevsky I. Dikopoltsev E. (2013). Neuronal D-Serine and glycine Release via the Asc-1 Transporter Regulates NMDA Receptor-dependent Synaptic Activity. J. Neurosci. 33, 3533–3544. 10.1523/JNEUROSCI.3836-12.2013 23426681 PMC6619521

[B41] Saji N. Niida S. Murotani K. Hisada T. Tsuduki T. Sugimoto T. (2019). Analysis of the Relationship between the Gut Microbiome and Dementia: a Cross-Sectional Study Conducted in Japan. Sci. Rep. 9, 1008. 10.1038/s41598-018-38218-7 30700769 PMC6353871

[B42] Savignac H. M. Corona G. Mills H. Chen L. Spencer J. P. Tzortzis G. (2013). Prebiotic Feeding Elevates central Brain Derived Neurotrophic Factor, N-Methyl-D-Aspartate Receptor Subunits and D-Serine. Neurochem. Int. 63, 756–764. 10.1016/j.neuint.2013.10.006 24140431 PMC3858812

[B43] Shi H. Yu Y. Lin D. Zheng P. Zhang P. Hu M. (2020). β-Glucan Attenuates Cognitive Impairment via the Gut-Brain axis in Diet-Induced Obese Mice. Microbiome 8, 143. 10.1186/s40168-020-00920-y 33008466 PMC7532656

[B44] Verdú E. F. Bercik P. Verma-Gandhu M. Huang X. X. Blennerhassett P. Jackson W. (2006). Specific Probiotic Therapy Attenuates Antibiotic Induced Visceral Hypersensitivity in Mice. Gut 55, 182–190. 10.1136/gut.2005.066100 16105890 PMC1856497

[B45] Wang C. Yu Q. Li D. Sun N. Huang Y. Zhang Y. X. (2021). Reduced D-Serine Release May Contribute to Impairment of Long-Term Potentiation by Corticosterone in the Perforant Path-Dentate Gyrus. Neurochem. Res. 46, 2359–2375. 10.1007/s11064-021-03380-4 34146194

[B46] Wang J. Cheng X. Zeng J. Yuan J. Wang Z. Zhou W. (2017). LW-AFC Effects on N-Glycan Profile in Senescence-Accelerated Mouse Prone 8 Strain, a Mouse Model of Alzheimer's Disease. Aging Dis. 8, 101–114. 10.14336/AD.2016.0522 28203484 PMC5287383

[B47] Wang J. Cheng X. Zhang X. Cheng J. Xu Y. Zeng J. (2016). The Anti-aging Effects of LW-AFC via Correcting Immune Dysfunctions in Senescence Accelerated Mouse Resistant 1 (SAMR1) Strain. Oncotarget 7, 26949–26965. 10.18632/oncotarget.8877 27105505 PMC5053624

[B48] Wang J. Lei X. Xie Z. Zhang X. Cheng X. Zhou W. (2019). CA-30, an Oligosaccharide Fraction Derived from Liuwei Dihuang Decoction, Ameliorates Cognitive Deterioration via the Intestinal Microbiome in the Senescence-Accelerated Mouse Prone 8 Strain. Aging (Albany NY) 11, 3463–3486. 10.18632/aging.101990 31160541 PMC6594795

[B49] Wang J. Liu Y. Cheng X. Zhang X. Liu F. Liu G. (2017). The Effects of LW-AFC on the Hippocampal Transcriptome in Senescence-Accelerated Mouse Prone 8 Strain, a Mouse Model of Alzheimer's Disease. J. Alzheimers Dis. 57, 227–240. 10.3233/JAD-161079 28222521

[B50] Wang J. Ye F. Cheng X. Zhang X. Liu F. Liu G. (2016). The Effects of LW-AFC on Intestinal Microbiome in Senescence-Accelerated Mouse Prone 8 Strain, a Mouse Model of Alzheimer's Disease. J. Alzheimers Dis. 53, 907–919. 10.3233/JAD-160138 27340848

[B51] Wang J. Zhang K. Chen X. Liu X. Teng H. Zhao M. (2017). Epigenetic Activation of ASCT2 in the Hippocampus Contributes to Depression-like Behavior by Regulating D-Serine in Mice. Front. Mol. Neurosci. 10, 139. 10.3389/fnmol.2017.00139 28536503 PMC5422558

[B52] Wang J. Zhang X. Cheng X. Cheng J. Liu F. Xu Y. (2017). LW-AFC, A New Formula Derived from Liuwei Dihuang Decoction, Ameliorates Cognitive Deterioration and Modulates Neuroendocrine-Immune System in SAMP8 Mouse. Curr. Alzheimer Res. 14, 221–238. 10.2174/1567205013666160603001637 27335033

[B53] Wang J.-H. Lei X. Cheng X.-R. Zhang X.-R. Liu G. Cheng J.-P. (2016). LW-AFC, a New Formula Derived from Liuwei Dihuang Decoction, Ameliorates Behavioral and Pathological Deterioration via Modulating the Neuroendocrine-Immune System in PrP-hAβPPswe/PS1ΔE9 Transgenic Mice. Alz Res. Ther. 8, 57. 10.1186/s13195-016-0226-6 PMC5154149 27964740

[B54] Wei M. Feng S. Zhang L. Wang C. Chu S. Shi T. (2021). Active Fraction Combination from Liuwei Dihuang Decoction Improves Adult Hippocampal Neurogenesis and Neurogenic Microenvironment in Cranially Irradiated Mice. Front. Pharmacol. 12, 717719. 10.3389/fphar.2021.717719 34630096 PMC8495126

[B55] Wolosker H. (2018). The Neurobiology of D-Serine Signaling. Adv. Pharmacol. 82, 325–348. 10.1016/bs.apha.2017.08.010 29413526

[B56] Zeng J. Cheng B. Huang Y. Zhang X. Wang C. Sun N. (2019). Active Fraction Combination from Liuwei Dihuang Decoction (LW-AFC) Alleviated the LPS-Induced Long-Term Potentiation Impairment and Glial Cells Activation in Hippocampus of Mice by Modulating Immune Responses. Evid. Based Complement. Alternat Med. 2019, 3040972. 10.1155/2019/3040972 31636681 PMC6766147

[B57] Zhou W. Cheng X. Zhang Y. (2016). Effect of Liuwei Dihuang Decoction, a Traditional Chinese Medicinal Prescription, on the Neuroendocrine Immunomodulation Network. Pharmacol. Ther. 162, 170–178. 10.1016/j.pharmthera.2016.02.004 26896567

